# Phylogenetic and Structural Diversity in the Feline Leukemia Virus *Env* Gene

**DOI:** 10.1371/journal.pone.0061009

**Published:** 2013-04-11

**Authors:** Shinya Watanabe, Maki Kawamura, Yuka Odahara, Yukari Anai, Haruyo Ochi, So Nakagawa, Yasuyuki Endo, Hajime Tsujimoto, Kazuo Nishigaki

**Affiliations:** 1 Laboratory of Molecular Immunology and Infectious Disease, The United Graduate School of Veterinary Science, Yamaguchi University, Yamaguchi, Japan; 2 Laboratory of Molecular Immunology and Infectious Disease, Joint Faculty of Veterinary Medicine, Yamaguchi University, Yamaguchi, Japan; 3 Department of Organismic and Evolutionary Biology, Harvard University, Cambridge, Massachusetts, United States of America; 4 Laboratory of Small Animal Internal Medicine, Joint Faculty of Veterinary Medicine, Kagoshima University, Kagoshima, Japan; 5 Department of Veterinary Internal Medicine, Graduate School of Agricultural and Life Sciences, The University of Tokyo, Tokyo, Japan; Centro Nacional de Microbiología - Instituto de Salud Carlos III, Spain

## Abstract

Feline leukemia virus (FeLV) belongs to the genus *Gammaretrovirus*, and causes a variety of neoplastic and non-neoplastic diseases in cats. Alteration of viral *env* sequences is thought to be associated with disease specificity, but the way in which genetic diversity of FeLV contributes to the generation of such variants in nature is poorly understood. We isolated FeLV *env* genes from naturally infected cats in Japan and analyzed the evolutionary dynamics of these genes. Phylogenetic reconstructions separated our FeLV samples into three distinct genetic clusters, termed Genotypes I, II, and III. Genotype I is a major genetic cluster and can be further classified into Clades 1–7 in Japan. Genotypes were correlated with geographical distribution; Genotypes I and II were distributed within Japan, whilst FeLV samples from outside Japan belonged to Genotype III. These results may be due to geographical isolation of FeLVs in Japan. The observed structural diversity of the FeLV *env* gene appears to be caused primarily by mutation, deletion, insertion and recombination, and these variants may be generated *de novo* in individual cats. FeLV interference assay revealed that FeLV genotypes did not correlate with known FeLV receptor subgroups. We have identified the genotypes which we consider to be reliable for evaluating phylogenetic relationships of FeLV, which embrace the high structural diversity observed in our sample. Overall, these findings extend our understanding of *Gammaretrovirus* evolutionary patterns in the field, and may provide a useful basis for assessing the emergence of novel strains and understanding the molecular mechanisms of FeLV transmission in cats.

## Introduction

Feline leukemia virus (FeLV) is an exogenous retrovirus, belonging to the genus *Gammaretrovirus*. FeLV has been shown to induce many diseases in cats, such as thymic lymphoma, multicentric lymphoma, myelodysplastic syndromes (MDS), acute myeloid leukemia (AML), aplastic anemia, and immunodeficiency [1], [2]. The outcome of natural FeLV infection is variable, and little is known about the mechanisms of pathogenesis. Variants of FeLV that induce specific types of disease have been described, and several characteristic genetic changes in the long terminal repeat (LTR), as well as the *env* gene in the viral genomes appear to be responsible for pathogenicity. For example, FeLV proviruses molecularly cloned from lymphomas typically contain two or three tandem direct repeats of enhancer elements in the LTR [3]–[5]. On the other hand, LTRs of FeLVs derived from non-neoplastic disease or weakly pathogenic strains contain only a single copy of the enhancer [4], [6]–[9], but may contain other repeated elements such as the upstream region of the enhancer (URE) in MDS [1] and AML [10], [11], and the 21-bp triplication in non-T-cell disease [12], [13].

In addition to characteristic LTR structures, *env* genes also appear to play a role in pathogenicity. For example, the *env* gene of an anemia-inducing FeLV variant (Sarma strain) contains mutations and recombination, and its pathogenicity results from the Env protein binding to and disrupting the cellular function of FLVCR1, which acts as a receptor for the strain [14]–[16]. Mutations of the *env* gene and unique receptor usage have also been identified in FeLV variants that cause immunosuppression in cats (feline acquired immunodeficiency syndrome) [7], [17]–[18], and several other studies have similarly linked disease outcome to determinants located in the *env* region [19], [20].

The primary translation product of the FeLV *env* gene is processed through proteolytic cleavage into two functional units: the surface protein (SU; gp70) and the transmembrane protein (TM; p15E). The entry of retroviruses into target cells is governed by the interaction of glycoproteins on the retroviral SU with specific cell surface receptors [21]. FeLV can be categorized into several FeLV subgroups based on their interference and host range properties: FeLV-A, FeLV-B, FeLV-C, FeLV-AC and FeLV-T [14], [16], [22]–[24]. In addition to these FeLV subgroups, our laboratory recently identified a novel FeLV subgroup (FeLV-D) which was generated by ERV-DC *env* transduction [25]. FeLV-A is the most common subtype, and other subtypes may have arisen from this variant. For instance, it has been shown that FeLV-B arose through recombination in the *env* region between FeLV-A and endogenous FeLV sequences (enFeLV) present in the feline genome [9], [26], and FeLV-C apparently also arose through deletion and mutation of the FeLV-A *env* gene [2], [27]. Since the initial discovery of FeLV in domestic cats in 1964 [28]–[29], the virus has also been isolated from wild cats such as the Florida panther [30] and the Iberian lynx [31]. Preventing FeLV infection in both domestic and wild cats is of considerable interest. In the present study, we investigated patterns of FeLV genetic diversity based on entire *env* gene sequences sampled from FeLV isolates throughout Japan, as well as *env* gene sequences of several additional FeLV isolates from Europe and the Americas. We identify genotypes encompassing the full range of FeLV structural diversity, which we consider to be reliable for evaluating the phylogenetic relationships of the virus. Overall, the findings of this study extend the understanding of *Gammaretrovirus* evolutionary patterns in the field, and may provide a useful basis for assessing the emergence of novel strains and understanding the molecular mechanisms of FeLV transmission in cats. Our results may also provide insights into disease alteration caused by mutations of the *env* gene.

## Materials and Methods

### Epidemiological Survey of the Prevalence of FeLV Infection

From March to October 2008, a total of 1770 EDTA-anticoagulated blood samples were collected from cats admitted at 47 private veterinary hospitals, one located in each prefecture of Japan. The samples were voluntarily submitted from veterinarians. For each cat, the age, sex, and primary complaint were recorded. The health profile of these cats has been partly described previously [32]. Blood samples were stored between −20 and −30°C prior to DNA extraction, and each of the 1770 samples was tested for serological evidence of FeLV infection by screening for the FeLV Gag antigen using a commercially-available test kit (SNAP FeLV/FIV combo kit; IDEXX Laboratories Inc., USA). A summary of the FeLV status, age, gender and other basic background data for the sample of cats is provided in Table 1 **.** Cats that had outdoor access at least once a week were included in the study.

**Table 1:** Profile and FeLV serological status of cats used in this study.

		Total	FeLV+(%)
Age (Years)	<1	219	12 (5.5)
	1	184	33 (17.9)
	2	166	36 (21.7)
	3	114	20 (17.5)
	4	93	17 (18.3)
	5	97	14 (14.4)
	6	96	12 (12.5)
	7	77	9 (11.7)
	8	70	6 (8.6)
	9	63	2 (3.2)
	10	97	8 (8.2)
	11	53	8 (15.1)
	12	63	7 (11.1)
	13	57	3 (5.3)
	14	45	5 (11.1)
	15	39	3 (7.7)
	>15	73	2 (2.7)
	unknown	164	19 (11.6)
	total	1770	216
Gender	Male (intact)	412	72 (17.5)
	Castrated Male	518	67 (12.9)
	Female (intact)	280	27 (9.6)
	Spayed Female	517	47 (9.1)
	unknown	43	3 (7.0)
	total	1770	216
Outdoor Access/week	1day	200	19 (9.5)
	∼4days	152	17 (11.2)
	∼6days	74	4 (5.4)
	everyday	1290	162 (12.6)
	unknown	54	14 (25.9)
	total	1770	216

### Detection and Amplification of FeLV Proviral DNA

Genomic DNA was isolated from blood samples testing positive for the FeLV antigen, using the QIAamp DNA Blood kit (QIAGEN, Tokyo, Japan) according to the manufacturer’s instructions. PCR primers for a full-length FeLV *env* amplification were designed from the conserved regions of *pol*, *env*, and LTR sequences of FeLV strains. PCR primers used were as follows: Fe-8S (5′-CATCGAGATGGAAGGTCCAACG-3′, position 5974–5995 of FeLV-A 61E (GeneBank M18247), Fe-4S (5′-TCCAACGCACCCAAAACCCTCT-3′, position 5989–6010 of FeLV-A 61E), Fe-3R (5′-CATGGTYGGTCYGGATCGTATTG-3′, position 7886–7908 of FeLV-A 61E), Fe-9S (5′-GAGACCTCTAGCGGCGGCCTAC-3′, position 5711–5732 of FeLV-A 61E), Fe-7R (5′-GTCAACTGGGGAGCCTGGAGAC-3′, position 8174–8195 of FeLV-A 61E) and PRB1 (5′-CTGTTCACTCCTCGACAACG-3′ [33]). The PCR primers used to amplify the c-*myc* exon 2 region were MY-1F (5′-GAGGAGGAGAACTTCTACCAGCA-3′) and MY-2R (5′-CTGCAGGTACAAGCTGGAGGT-3′).

PCR was performed using approximately 100 ng genomic DNA in 50 µL reactions, with PrimeSTAR HS DNA Polymerase enzyme (Takara, Japan). PCR cycling conditions were as follows: 98°C for 10 s, 62°C for 5 s, 72°C for 1.5 min, for 30 cycles, followed by 5 min at 72°C. PCR products were directly cloned into the pCR4Blunt-TOPO vector (Invitrogen) or the pUC118 vector (Mighty cloning kit, Takara), and these recombinants were also used as a plasmid library to isolate full-length FeLV-B *env* genes and *env* deletion mutants. For detection of FeLV-B *env*, PCR was performed as described above, using primers PRB1 and Fe-3R. This primer pair is specific for FeLV-B, and a full-length FeLV-B *env* was isolated by screening the *env* plasmid library with colony PCR methods using these two primers. Based on the entire *env* gene, non-recombinant virus, recombinant virus including FeLV-B (identified by PCR) and non-FeLV-B (identified by recombination analysis), and deletion type and/or insertion type virus were isolated from the plasmid libraries using primer pairs Fe-8S/4S and Fe-3R. Alternatively, the entire *env* gene was amplified with primers Fe-9S and Fe-7R (specific for *pol* and exogenous LTR U3 regions), and these fragments were cloned into the pCR4Blunt-TOPO vector (Invitrogen). PCR was performed on a TaKaRa PCR Thermal Cycler Dice (Takara, Japan), and plasmids were sequenced with an Applied Biosystems 3730xl DNA Analyzer (Applied Biosystems, USA).

### Recombination Analysis

The Simplot software (version 3.5.1) was initially used to perform similarity and boot scanning analyses of a query sequence against a set of other sequences [34], [35]. This method measures the similarity/dissimilarity of the query sequence to a set of reference sequences.

### Phylogenetic Analysis

The *env* sequences were aligned using Muscle [36] and these initial alignments were manually edited by eye in SEAVIEW [37]. FeLV *env* sequences from the NCBI database (see Table S1) were also included in the alignments. Subsequent phylogenetic analyses were based on virtually complete sequences of the SU and TM regions within the *env* gene (position 6080–7885 of FeLV-A 61E). Recombinant *env* genes, including FeLV-B identified by recombination analyses, were usually excluded from the data set for the purposes of phylogenetic reconstruction. However, partial sequences from some recombinants were included in the analyses after excluding their recombinant regions to determine FeLV genotype from cats (ST19, IT10, WY24, SA15 and IT38), from which non-recombinants were not identified. Partial sequences from representatives of the FeLV-C and FeLV-B subgroups were also used for the analyses. Phylogenetic trees were estimated using both the maximum likelihood method (ML) [38] and the neighbor joining approach (NJ) [39], and sequences from endogenous FeLVs were used as outgroups to root the trees. ML reconstructions were performed in PhyML [40], applying the general time-reversible (GTR) model of nucleotide substitution. Nodal support was assessed via a likelihood-ratio test (LRT) [41], [42], as implemented in PhyML. NJ trees were constructed using CLUSTALW [43], [44], and distances were calculated using the Kimura two-parameter (K2P) method [45]. To assess the support for nodes in the NJ trees, a bootstrap analysis was performed using 1000 pseudoreplicates. Phylogenetic trees were visualized using MEGA5 [46], [47].

### Viral Interference Assay

Construction of FeLV *env*-expression plasmids and performance of viral interference assays using GPLac cells have been described elsewhere [25]. FeLV *env* fragments subcloned in pCR4Blunt-TOPO were *Eco*RI-digested, and FeLV *env* genes in pUC118 were PCR-amplified using primers Fe-44S and Fe-50R (5′-CGGAATTCATCGAGATGGAAGGTCC-3′ and 5′-TTGAATTCTCATGGTTGGTCTGGATCGTATTG-3′ respectively). The restriction sites for *Eco*RI are underlined. These products were cloned into pFUΔss expression plasmid. GPLac cells, which contain MLV *gag-pol* and pMXs-nls*Lac*Z-IRES-*Puro^r^* (pMXs-nLIP) retroviral reporter, were transfected with FeLV *env*-expressing plasmids to produce *Lac*Z-carrying pseudotype viruses. After selection of the cells with 200 µg/ml Zeocin and 1 µg/ml Puromycin for >2 weeks, filtered supernatants were used for viral interference assays. HEK293T [48], [49] or AH927 cells [50] persistently infected with FeLV-A clone33 [10], FeLV-B GA (Gardner-Arnstein) strain [51], FeLV-C Sarma strain [8], or non-infected cells were cultured in Dulbecco’s Minimal Essential Medium supplemented with 10% fetal calf serum (FCS). These cells were infected with each pseudotype virus in the presence of polybrene (4 µg/ml) for 2 days, and then stained with X-gal (5-bromo-4-chloro-indolyl-β-D-galactopyranoside). Microscopic visualizations of the results were saved as digital images, and blue-stained nuclei were counted as infectious units (I.U.).

### Analysis of Branch-specific Selection Forces

For these phylogenetic analyses, we utilized *env* gene sequences that meet the following criteria: a) have no recombination with enFeLV; b) are longer than 1800-bp; c) do not contain any frame shift mutations or undetermined nucleotides. We then translated these sequences and computed a multiple alignment at the amino acid level using the L-INS-i module of the MAFFT program [52]. Applying this amino acid alignment, we generated a corresponding nucleotide sequence alignment using TranslatorX [53]. Based on the multiple alignment of nucleotide sequences, we performed phylogenetic tree reconstruction using ML methods as implemented in RAxML v. 7.2.6 [54], applying the GTR model with a discrete gamma distribution to account for heterogeneity in evolutionary rates among sites (+G), and including an estimation of the proportion of invariant sites (+I). The robustness of the phylogenetic tree was evaluated by rapid bootstrapping [55] with 1000 pseudoreplicate datasets. To detect branch-specific positive selections, we used ML methods implemented in the codeml program of the PAML v. 4.5 package [56] to calculate the ratio of substitution rates at non-synonymous and synonymous sites (dN/dS) for the each branch of the phylogenetic tree. We applied the F3×4 codon frequency model in which the average nucleotide frequencies are calculated separately at the three codon positions [57].

### Ethics of Experimentation and Research

All experiments were approved by Genetic Modification Safety Committee of Yamaguchi University (Permit Number: J12022).

### Nucleotide Sequence Accession Numbers

The nucleotide sequences reported in this paper are available in the DDBJ, EMBL, and GenBank nucleotide sequence databases under the following accession numbers: AB635483–AB635582, AB635586–AB635685, AB635687–AB635763, AB635764–AB635863 and AB672612.

## Results

### Prevalence of FeLV in Japan

**Figure 1:**
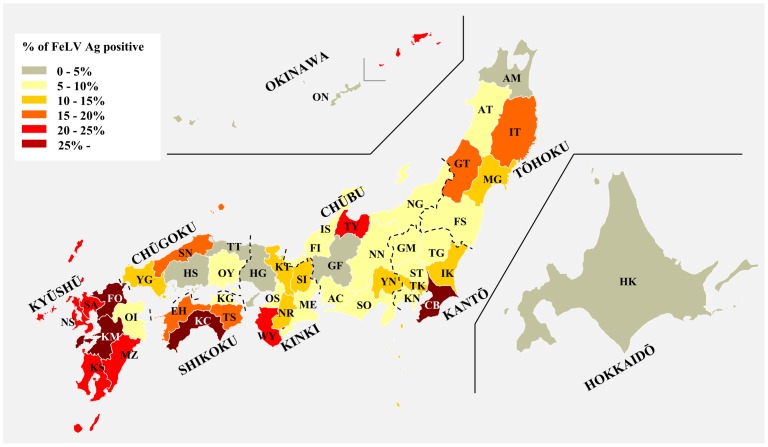
Incidence of FeLV in blood samples collected from private veterinary hospitals located in each prefecture of Japan. The incidence of samples testing positive for the FeLV antigen was divided into six color-coded groups in increments of 5%. A two-letter code was assigned to each prefecture as described in Table S2.

A clinical and molecular-based epidemiological survey of FeLV infection covering the whole of Japan was performed in this study. FeLV Gag antigen-positive reactions were detected in 216 (12.2%) of the 1770 cats from which blood samples were collected (Table 1), and infected cats were identified in 45 of the 47 private veterinary hospitals visited. As shown in Fig. 1 and Table S2, The incidence of FeLV was higher in southern areas of Japan, such as parts of the Kyūshū area (Fukuoka, Saga, Nagasaki, Kumamoto, Miyazaki and Kagoshima prefectures; >20% positive) and parts of the Shikoku area (Ehime, Tokushima, and Kōchi prefectures; >15% positive). The seroprevalence of FeLV infection in Iwate, Yamagata, Chiba, Toyama, Wakayama, and Shimane prefectures was also high (>15% positive) in comparison to the average FeLV infection rates throughout Japan (12.2%). Although all owned cats we tested had outdoor access at least once a week, there was no clear correlation between FeLV infection and the number of days of outdoor access (Table 1). Interestingly, of the 286 cats vaccinated against FeLV, 18 tested positive for the virus (6.3%) (Table 1). Overall, these results indicate that FeLV infection is observed widely in Japan, and the control and prevention of this infectious disease should thus be a focal point for Japanese veterinary medicine.

### Detection of Proviral *env* Fragments by PCR

**Figure 2:**
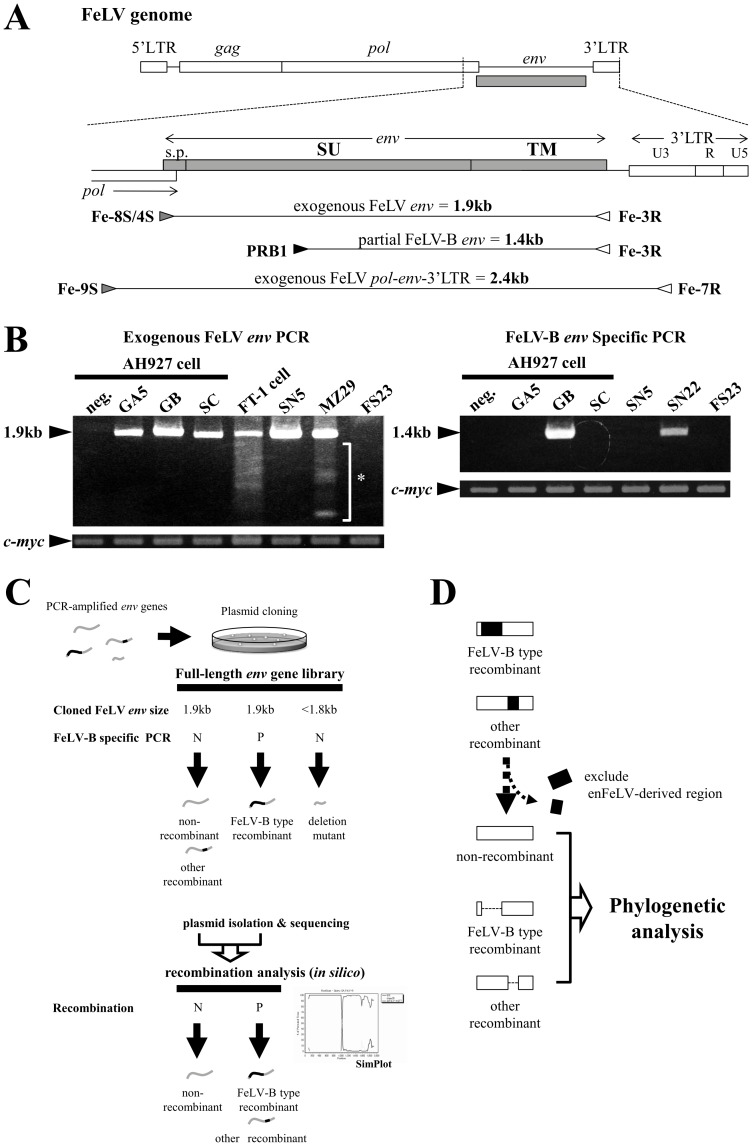
Detection of FeLV *env* genes by PCR, and strategies for analysis of these genes. (A) Strategy used for generating PCR products. Schematics of coding sequences for the FeLV *env* gene are shown. FeLV proviral *env* sequences were amplified by PCR with the primer pairs Fe-8S/Fe-4S and Fe-3R, PRB-1 and Fe-3R, Fe-9S and Fe-7R. The lengths of the expected products from amplifications using each primer pair are shown. The abbreviations s.p., SU and TM represent, respectively, signal peptide, surface glycoprotein, and transmembrane subunit. (B) The DNA templates for PCR amplifications were as follows: neg. (genomic DNA isolated from FeLV-negative AH927 cells), GA5 (DNA from FeLV-A Glasgow-1-infected AH927 cells), GB (DNA from Gardner-Arnstein FeLV-B-infected AH927 cells), SC (DNA from FeLV-C Sarma-infected AH927 cells), DNA from FT-1 cell line, DNA from sample SN5, DNA from sample MZ29, DNA from a FeLV-positive cat (SN22), and DNA from a FeLV-negative cat (FS23). c-*myc* was amplified as a positive control [25]. PCR products were electrophoresed and stained with ethidium bromide. Asterisk indicates atypical bands of *env* gene. (C) Each detected PCR fragment was cloned into a cloning plasmid, and full-length *env* gene libraries were constructed. Several unique *env* genes were isolated from these full-length *env* gene libraries using information on fragment size, or by screening using FeLV-B specific PCR. In addition to non-recombinant *env* genes, FeLV-B-type and other recombinants were isolated and analyzed. Furthermore, *env* genes smaller than full length were also analyzed. N indicates negative and P positive for FeLV-B detection or recombination detection. (D) For the most part, only non-recombinant sequences were used for phylogenetic analysis, but where recombinant sequences were included, their endogenous-derived regions were removed from the alignment. However, partial sequences from some recombinants as well as representatives of the FeLV-C and FeLV-B subgroups were included in the analyses after excluding their recombinant regions, to determine the FeLV genotypes from cats from which non-recombinants were not identified.

To evaluate the divergence of the FeLV *env* gene across Japan, we established a PCR method to specifically detect exogenous FeLV *env* genes (Fig. 2A, B). PCR amplification using primer pairs Fe-8S/Fe-4S and Fe-3R encompassed the entire FeLV *env* gene, and resulted in a fragment of predicted size (approximately 1.9 kb) when applied to AH927 cells infected with FeLV-A Glasgow-1 (GA5), FeLV-B Gardner-Arnstein (GB) or FeLV-C Sarma (SC) (Fig. 2B), and when applied to FT-1 cell lines [5] derived from the thymic lymphoma of a FeLV-positive cat, and samples of FeLV antigen-positive blood (SN5, MZ29). However, the 1.9 kb fragment was not detected in genomic DNA derived from uninfected AH927 cells, or in FeLV antigen-negative feline blood (FS23). This indicates that the primers did not amplify detectable levels of enFeLV, and that these two primer pairs are specific for *env* sequences of known FeLV-A, -B, and -C types (Fig. 2B). The sensitivity of this PCR method for detection of the *env* gene was investigated by application to the FeLV-A clone33 plasmid, and the limit of detection was determined to be 10 fg per reaction in this system (data not shown).

FeLV *env* PCR fragments were detected in 167 of the 216 samples of genomic DNA from FeLV antigen-positive cats. We were unable to amplify FeLV *env* fragments in the remaining seropositive samples, and in these samples we also failed to amplify LTR fragments despite attempts with different primer combinations, increased DNA concentration, lower annealing temperature and different DNA polymerase enzymes. These negative results may reflect divergent proviral sequences in primer sites for viral detection, low viral load, or other factors.

In addition to the fragments discussed above, several smaller fragments were amplified by the PCR primer pairs Fe-8S/Fe-4S and Fe-3R. For example, several smaller bands in addition to the expected 1.9-kb band were detected in FeLV-positive blood (MZ29) (Fig. 2B), and such bands were seen in 18 of the 167 PCR-positive samples. Eight of these 18 samples were from cats with neoplastic disease, including five lymphomas and three suspected cases of lymphoma. These fragments were cloned and their identities confirmed by sequencing.

A FeLV-B-specific detection system was established by PCR amplification using the enFeLV-specific PRB1 primer paired with an exogenous FeLV-specific antisense primer, Fe3R. These PCR reactions resulted in fragments with the predicted size of approximately 1.4 kb when applied to AH927 cells infected with FeLV-B, and FeLV antigen-positive blood (SN22), but not when applied to FeLV-A Glasgow-1 (GA5), FeLV-C Sarma (SC), FeLV antigen-positive (SN5) or negative blood (FS23) (Fig. 2A, B). Sequencing was used to confirm the identity of PCR fragments that were amplified with FeLV-B-specific primers, indicating that the primers did not amplify detectable levels of enFeLV, and that these primer pairs detected at least *env* sequences from the known FeLV-B types. FeLV-B *env* was present in 74 of the 167 (44.3%) samples of genomic DNA from FeLV antigen-positive cats in which the *env* gene had been detected via PCR. Our results regarding the incidence of FeLV-B infection were similar to results previously reported [2], [58], [59]. Our PCR method did not detect FeLV-B *env* fragments in any of the remaining samples from FeLV antigen-positive cats.

### Phylogenetic Analysis of FeLV *env* Gene Sequences

**Figure 3:**
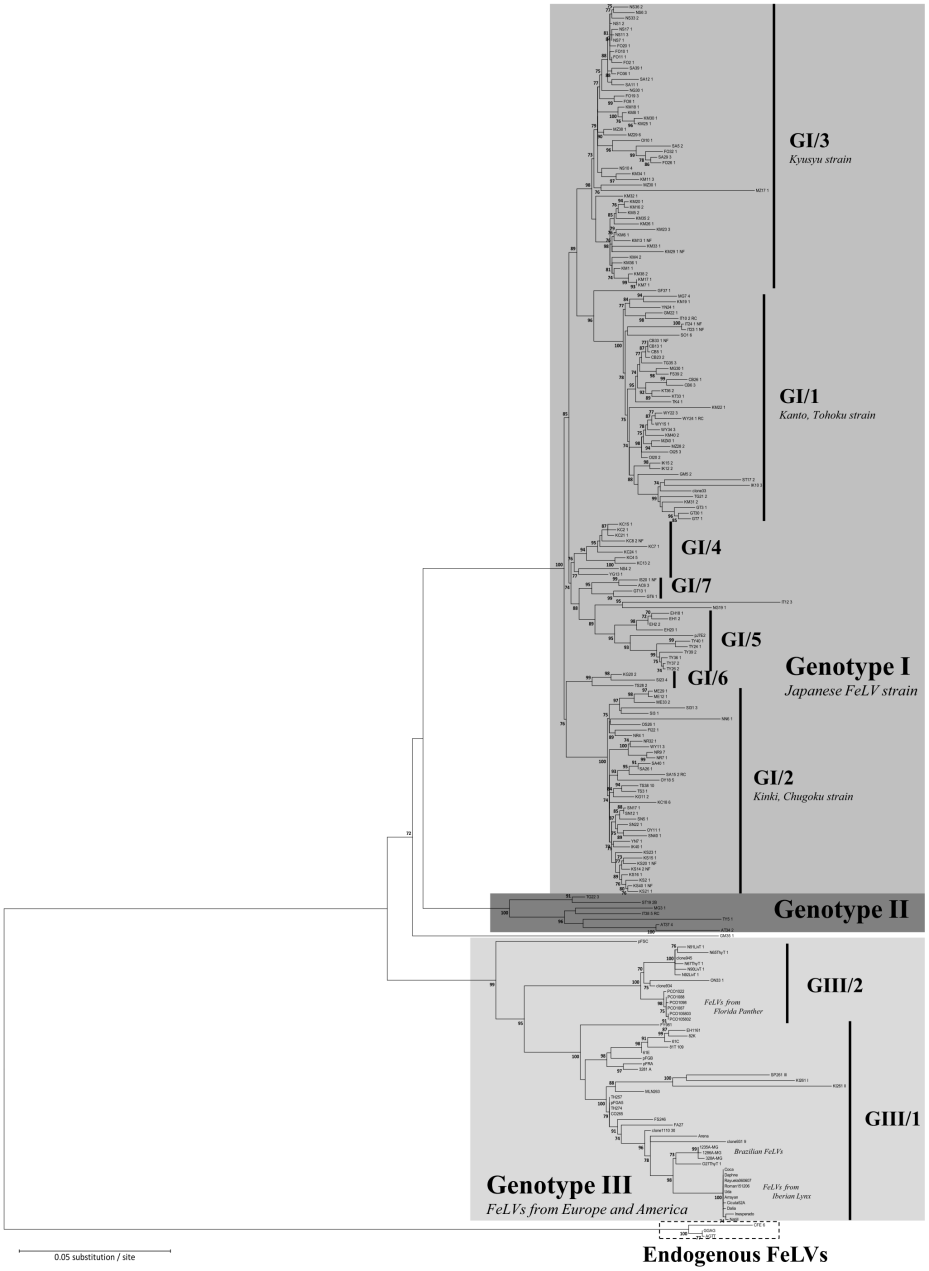
The best maximum-likelihood (ML) tree from phylogenetic analysis of near-full-length *env* nucleotide sequences generated in this study and obtained from the NCBI database. FeLV sequence information obtained from the NCBI database is listed in Table S1.

We isolated 261 clones of non-recombinant full-length *env* genes having an approximately 1.9 kb *env* gene. From the cases positive for FeLV-B and having an approximately 1.9 kb *env* gene, we isolated 76 FeLV-B specific clones, which were FeLV-B-positive, as determined by detecting a 1.4-kb fragment of partial FeLV-B *env* gene by our PCR method. In addition, from 18 cats we isolated 39 clones having several deletions of variable sizes. All of these clones were sequenced and analyzed. For the purposes of phylogenetic analysis, recombinant *env* genes such as FeLV-B, and *env* genes with large deletions or insertions, were identified by recombination analyses and excluded from the alignment (Fig. 2C). However, sequences from cats ST19, IT10, WY24, SA15 and IT38, (FeLV-C Sarma strain and FeLV-B GA strain), which are recombinant *env* genes, were included in the multiple sequence alignment after removal of their recombinant regions (Fig. 2D). The results from the phylogenetic analysis of the alignment using the ML method are shown in Fig. 3. ML and NJ methods both retrieved similar phylogenetic topologies (data not shown), in which all isolates (with the exception of GM35) grouped into three major, strongly supported clades: Genotype I (GI), Genotype II (GII), and Genotype III (GIII). Bootstrapping was employed to assess the support for these major groups and their component subclades, and we generally considered clades with bootstrap support values of >90% to be robust. Within Genotype I, seven clades were recognized as separate strains (see Fig. 3): GI/Clade1 (41 sequences), GI/Clade2 (37 sequences), GI/Clade3 (51 sequences), GI/Clade4 (10 sequences), GI/Clade5 (11 sequences), GI/Clade6 (3 sequences), and GI/Clade7 (4 sequences). Of these, all but one had bootstrap values <90%; Clade 4 was supported by a bootstrap value of only 76%, and this clade may thus be re-classified based on future analyses. FeLV-A clone33 [10] and pJ7E2 [5] previously isolated in Japan (obtained from the NCBI database) clearly belong within Genotype I. Several clones with unusually long branches were identified in the various clades (e.g., MZ17, IK10, NN6-1, SA15-2, SI31-3). These viruses appear to possess different evolutionary dynamics from the other samples. In addition to having long branches, IT12-3 and NG19-1 did not fit into any of the clades in Genotype I. Sequence GF37-1, though not possessing a long branch, was somewhat divergent from the Genotype I clades and also did not fit into any of these groupings.

Genotype II consisted of samples from only seven cats, and future large-scale analyses are likely to provide additional resolution of relationships in this group. Genotypes I and II were derived exclusively from Japan and were newly characterized in this study, while all FeLV strains from Europe, South America and the USA (obtained from the NCBI database) were classified as Group III. Genotype III corresponds to known FeLV strains such as FeLV-A clone 945 [60], FeLV-A 61E [6], FeLV-T 61C [7], FeLV-A Glasgow-1 [9], FeLV-A Richard (pFRA) [61], FeLV-AC FY981 [62], FeLVs derived from Iberian lynx [31], Brazilian FeLVs [58], and the exogenous FeLV components of the FeLV-C Sarma and FeLV-B GA strains. Most Genotype III samples were included in two strongly supported groups, GIII/Clade1 and GIII/Clade2. Sequences from a single cat (ON33) collected in Okinawa (ON) prefecture, Japan were classified as GIII/Clade2. Interestingly, GM35 sequences from Gunma (GM) prefecture did not fall within any of the major genotypes. In this analysis, there were no GI or GII viruses detected outside of Japan, suggesting that our genotypic classification may be in some way dependent on geographical distribution.

We did not find any cases of superinfection with multiple FeLV groups in any cats; all clones obtained from a given individual belonged to the same clade. Furthermore, mutant *env* containing insertions or deletions, isolated from individual cats, had a high level of homology with full-length *env* derived from the same individual, suggesting that such mutants possibly occur *de novo* in these individuals. Our results suggest that FeLV has perhaps undergone extensive evolutionary diversification within Japan, and the genetic distinctness of Japanese FeLV strains may indicate a long history of geographical isolation in the country.

### Receptor Usage of Representative FeLV *env* Genes from Japan

Next, to delineate FeLV subgroups on the basis of receptor usage, we conducted a viral interference assay. *Env* genes from each genotype and clade were inserted into the mammalian expression vector, which was introduced into GPLac cells [25] to produce FeLV *env* pseudotype viruses carrying the *Lac*Z marker gene. HEK293T cells persistently infected with FeLV-A clone33, FeLV-B GA and FeLV-C Sarma were superinfected with each pseudotype virus. As shown in Table 2, the superinfection of 12 clones (two from GI/1, three from GI/2, one from GI/4, two from GI/5, and one each from GI/6, GI/7, GI/unclassified and GII) was completely interfered by FeLV-A clone33, but not by FeLV-B and FeLV-C receptor subgroups. This indicates that these Japanese isolates belong in the FeLV-A subgroup and possess the same receptor usage as FeLVs in other countries despite their different genotypic classification. Furthermore, pseudotype viruses from NS33-4 (GI/3) and KC18-6 (GI/2) were unable to infect uninfected HEK293T cells, HEK293T cells persistently infected with FeLV-A clone33 (HEK293T/FeLV-A clone33) or cells infected with FeLV-C Sarma (HEK293T/FeLV-C). However, these pseudotype viruses could infected HEK293T cells infected with FeLV-B GA (HEK293T/FeLV-B) (Table 2). It has previously been reported that the FeLV-T subgroup can infect cells infected with FeLV-B [17]. In order to determine whether NS33-4 and KC18-6 viruses belong to the FeLV-T subgroup, the supernatant of 3201 cells as a source of a co-factor, FeLIX, for FeLV-T was used in an additional viral infection assay. As shown in Table 3, both KC18-6 and NS33-4 were able to infect AH927 cells and HEK293T cells in the presence of the conditioned medium, but not in its absence. These FeLV clones had mutations within their SPHQ motifs which are related to the unique receptor usage of FeLV-T [17], suggesting that both KC18-6 and NS33-4 belong to the FeLV-T subgroup. Taken together, our results suggest that most of the FeLV isolates distributed in Japan may show the same receptor usage as FeLVs in other countries despite their different genotypes.

**Table 2:** Viral interference assay using *Lac*Z pseudotyped viruses derived from FeLV *env* genes.

env pseudotypes		HEK293T cells preinfected with			
		No virus	FeLV-A	FeLV-B	FeLV-C
			clone33	GA	Sarma
	vector	<1	<1	<1	<1
	FeLV-A(Glasgow1)	7.80×10^2^	<1	6.50×10^2^	6.50×10^2^
	FeLV-B (GA)	3.28×10^5^	4.28×10^5^	<1	4.06×10^5^
	FeLV-C (Sarma)	7.80×10^2^	7.80×10^2^	1.04×10^3^	<1
GI/1	CB13-1	3.90×10^2^	<1	2.60×10^2^	5.20×10^2^
GI/1	MZ40-1	7.80×10^2^	<1	2.08×10^3^	2.60×10^3^
GI/2	KS16-1	2.21×10^3^	<1	1.82×10^3^	2.08×10^3^
GI/2	ME12-1	2.08×10^3^	<1	3.51×10^3^	3.25×10^3^
GI/2	NN6-1	1.30×10^4^	<1	4.47×10^4^	3.97×10^4^
GI/4	KC2-1	5.07×10^3^	<1	3.25×10^3^	4.16×10^3^
GI/5	TY26-2	1.30×10^3^	<1	1.17×10^3^	1.95×10^3^
GI/5	EH2-2	2.99×10^3^	<1	5.20×10^2^	1.56×10^3^
GI/6	KG20-2	6.24×10^3^	<1	1.17×10^3^	1.95×10^3^
GI/7	AC6-3	9.10×10^2^	<1	1.17×10^3^	1.56×10^3^
GI	NG19-1	1.17×10^3^	<1	9.10×10^2^	1.30×10^3^
GII	TG22-3	2.99×10^3^	<1	1.17×10^3^	3.25×10^3^
GI/3	NS33-4	<1	<1	5.89×10^3^	<1
GI/2	KC18-6	<1	<1	6.07×10^2^	<1
					Titer: I.U./ml

The data indicate an average of three independent experiments.

**Table 3:** Viral interference assay using *Lac*Z pseudotyped viruses derived from FeLV *env* genes.

*env*pseudotypes	Motif	AH927cells with		HEK293Tcells with	
	SPHQ	Medium	3201Sup.	medium	3201 Sup.
vector	–	<1	<1	<1	<1
FeLV-A(Glasgow1)	SPHQ	6.15×10^3^	5.81×10^3^	n.d.	n.d.
FeLV-B(GA)	SPHQ	2.08×10^3^	6.07×10^2^	1.22×10^4^	5.29×10^3^
KC18-6	SPPQ	<1	2.69×10^3^	<1	2.43×10^3^
NS33-4	GPPQ	<1	9.53×10^3^	<1	2.69×10^3^
					Titer: I.U./ml

### Geographic Distribution of Each FeLV Clade

**Figure 4:**
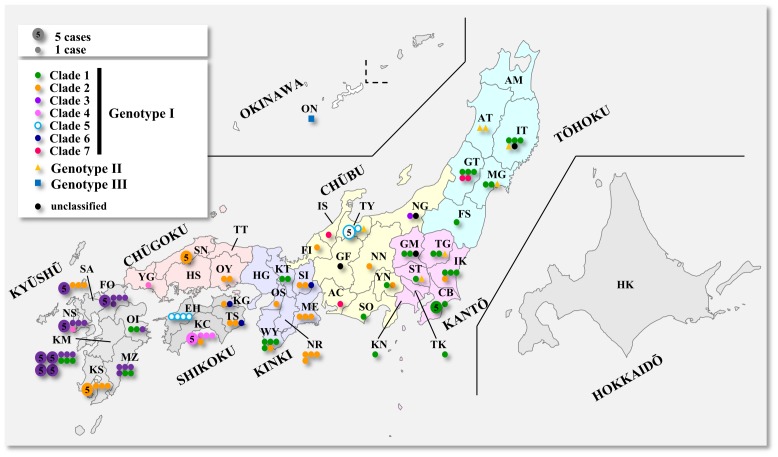
Geographic distribution of the major FeLV genotypic groups (I, II, III) and the seven clades of Genotype I. Each color-coded dot represents one infected cat (small dot) or 5 infected cats (large dot). Colored areas indicate provinces of Japan such as Hokkaidō, Tōhoku, Kantō, Chūbu, Kinki, Chūgoku, Shikoku and Kyūshū. A two-letter code was assigned to each prefecture as described in Table S2. A detailed summary of the geographic distribution of all groups is provided in Table S3.

We plotted Japanese viral isolates on a map to investigate the relationship between geographical distribution and reconstructed phylogenetic patterns. As shown in Fig. 4 and Table S3, GI/Clade1 and GI/Clade2 are broadly distributed in Japan, while GI/Clade1 is more concentrated in the Tōhoku and Kantō regions, and GI/Clade2 is more concentrated in the Kinki and Chūgoku regions. GI/Clade3 tends to be distributed in the Kyūshū region. GI/Clade4 samples are found predominantly in Kōchi (KC) prefecture (Shikoku region) and GI/Clade5 occur in the prefectures of Toyama (TY)(Chūbu region) and Ehime (EH) (Shikoku region) as they appear in Fig. 4 and Table S3. Most phylogenetic groupings are thus broadly consistent with geographic distribution, indicating that the major genotypes and clades may have arisen in allopatry within the areas of their current occupancy, or that FeLVs were introduced several times into Japan via distinct transmission routes. Interestingly, GI/Clade1 representatives in the Wakayama (WY) prefecture (Kinki region) and the Kyūshū region are substantially isolated from each other geographically despite their close phylogenetic relationship. This could plausibly be the result of an artificial transmission via anthropogenic transportation of infected cats. We also analyzed the fixation and purifying selection of each genotype and clade by calculating the branch-specific ratios of substitutions at non-synonymous and synonymous sites (dN/dS) (see Table S4 for a list of the sequences used). The dN/dS ratio for the root branch of each genotype and clade is shown on a schematic representation of the best phylogenetic tree from ML analysis of the complete nucleotide alignment (see Fig. S1 and Fig.S2). In most cases dN/dS values were less than 1, providing no evidence of positive selection. However the clade containing IT12-3 and NG19-1 had a dN/dS ratio of 1.0322, suggesting that selection may be in operation in this lineage.

### Recombination Analysis

**Figure 5:**
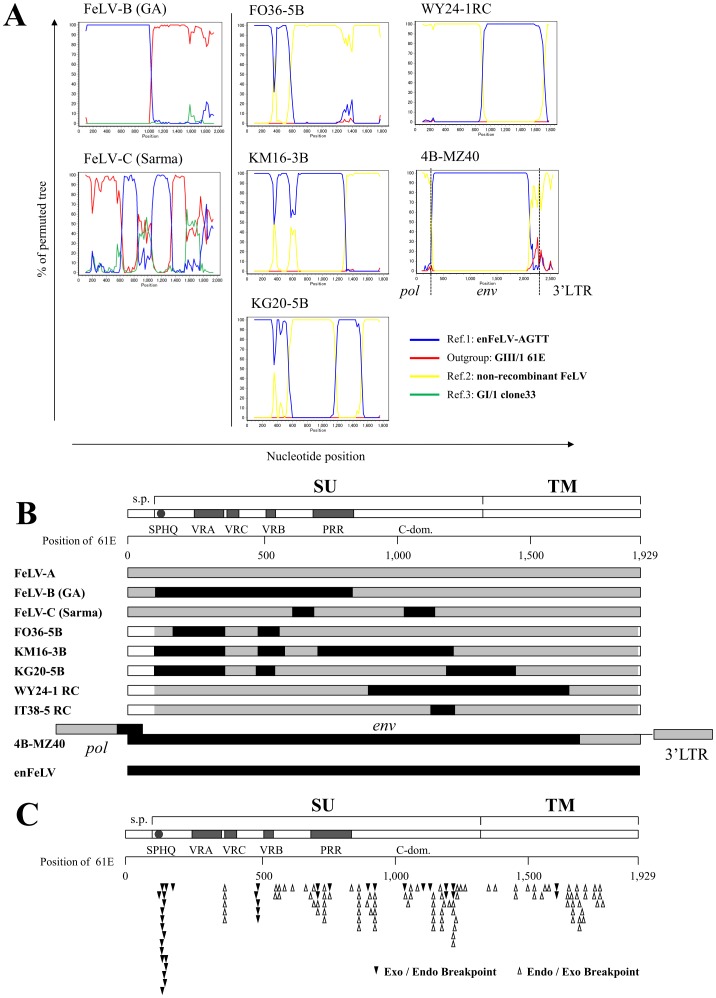
Analyses of FeLV *env* gene recombination. (A) Plots of similarity between a set of indicated sequences. Each curve is a comparison between the title sequence and the color-coded reference FeLV *env* sequences. The horizontal axis indicates physical position along the *env* sequences, and the vertical axis indicates % of the permuted tree. Non-recombinant FeLV was derived from a similar case that had each indicated recombinant except for WY24-1RC. Non-recombinant WY22-3 was used as a reference for WY24-1RC. (B) Schematic representation of the various recombination structures identified using similarity plot analysis. The motifs are abbreviated s.p. (signal peptide), SPHQ (SPHQ motif), VRA (variable region A), VRC (variable region C), VRB (variable region B), PRR (proline-rich region) and C-dom. (C-terminal domain). (C) Positions of recombination (breakpoints) in *env* genes from 80 recombinant clones (76 belonging to the FeLV-B subgroup, and 4 non-typical recombinants). Breakpoints of 5′ exogenous and 3′ endogenous *env* sequences, and 5′ endogenous and 3′ exogenous *env* sequences are indicated on the FeLV-A 61E sequence.

FeLV-B *env* sequences contain enFeLV *env* sequences as a result of recombination. To determine structural diversity of FeLV-B, full-length FeLV-B-like *env* sequences were isolated from plasmid libraries constructed using Fe-8S and Fe-3R primers, which amplify exogenous FeLV *env* genes (Fig. 2C). FeLV-B and other recombinant sequences isolated from PCR were subjected to similarity plot analyses following boot scanning analyses to identify recombination events between exogenous FeLV and enFeLV sequences (Fig. 2C, D). The recombinant *env* sequences were compared with non-recombinant exogenous FeLV *env* sequences derived from an identical sample or from the same clade, as well as with enFeLV-AGTT (endogenous FeLV), using FeLV-A clone33 or FeLV-A 61E as an outgroup. Similarity plot analysis (Fig. 5A) showed that all of FeLV-B-like *env* sequences arose from the recombination of exogenous FeLV *env* sequences and the endogenous FeLV-AGTT. According to the boot scanning analyses, recombination apparently produced the FeLV-B Gardner-Arnstein (GA) strain by uniting a section of 5′ endogenous FeLV *env* sequence and a section of 3′ exogenous FeLV *env* sequence. Furthermore, the FeLV-C Sarma strain was observed to be a product of recombination events in two regions of the *env* gene.

Fig. 5B shows representations of the recombination patterns that were observed in the present study. Many cases of recombination shared similarities with the recombination pattern in the FeLV-B GA strain (Fig. 5B). However, some unique recombination patterns were also observed. For example, clones FO36-5B, KG20-5B and KM16-3B contain both the VRA and VRB motifs from endogenous FeLV *env* sequences. Clones KG20-5B and KM16-3B possess endogenous FeLV sequences within the C-domain, and in KM16-3B the endogenous component extends to the proline-rich region (PRR). Interestingly, four clones (SA15-2 RC, WY24-1 RC, IT38-5 RC and IT10-2 RC) isolated from non-typical FeLV-B *env* sequences had only a 3′ portion of endogenous FeLV *env* sequence, whilst the 5′ region of their *env* sequences (the receptor binding domain, RBD), were apparently derived from FeLV-A (Fig. 5B and data not shown). Some *env* sequences (e.g. MZ40-5B) were almost entirely derived from endogenous FeLV *env*, and in these cases the boot scanning analysis did not detect recombination breakpoints between exogenous and endogenous FeLV sequences. To further confirm recombination in our virus samples, we isolated the 4B-MZ40 clone that possesses a portion of 3′ *pol*, a full-length endogenous *env* gene, and an exogenous LTR U3 section, by PCR amplification with specific primers, Fe-9S and Fe-7R (Fig. 2A), indicating with certainty that the recombinant virus was derived from exogenous FeLV (Fig. 5A, 5B). Interestingly, VRA recombination was often coupled with VRB recombination, as shown in Fig. 5B. When 5′ and 3′ recombination breakpoints were marked on the corresponding positions of the FeLV 61E *env* sequence, some recombination hot spots were observed and breakpoints tended towards regions of high homology between endogenous and exogenous FeLV sequences (Fig. 5C). However, recombination junctions varied substantially between different recombinant clones, indicating that multiple recombination events have occurred, producing appreciable structural diversity of the FeLV *env* gene. Furthermore, many recombinant viruses have apparently arisen *de novo* in individual cats. In order to determine whether recombinant viruses isolated in Japan belong to the FeLV-B receptor-usage phenotype, we conducted a viral interference assay using pseudotype viruses. Four of these (MZ40-5B, FO36-5B, IT38-5RC and WY24-1RC) were able to infect AH927 cells, and we tested whether they could also infect AH927/FeLV-A cells, AH927/FeLV-B cells and AH927/FeLV-C cells. As shown in Table 4, MZ40-5B and FO36-5B isolates could not infect AH927/FeLV-B cells, but were able to infect AH927/FeLV-A and AH927/FeLV-C cells, indicating that these two viruses belong to the FeLV-B receptor-usage subgroup. On the other hand, IT38-5RC and WY24-1RC isolates (which contain enFeLV sequences in the TM region and/or the C-domain) were unable to infect AH927/FeLV-A cells, but could infect AH927/FeLV-B and AH927/FeLV-C cells. These isolates must thus belong to the FeLV-A subgroup despite the fact that they are clearly recombinant in nature.

**Table 4:** Pseudotype viruses derived from FeLV recombinant *env* genes having endogenous FeLV sequences were tested for viral interference assay.

*env* pseudotypes	Recombination					AH927 cells preinfected with			
	SU				TM	No virus	FeLV-A	FeLV-B	FeLV-C
	VRA	VRC	VRB	C-dom.			clone33	GA	Sarma
vector	–	–	–	–	–	<1	<1	<1	<1
FeLV-A (Glasgow1)	N	N	N	N	N	1.39×10^5^	<1	1.03×10^5^	6.92×10^4^
FeLV-B (GA)	R	R	R	N	N	3.29×10^3^	2.08×10^3^	<1	1.82×10^3^
FeLV-C (Sarma)	N	N	N	R	N	6.93×10^2^	1.13×10^3^	1.04×10^3^	<1
MZ40-5B	R	R	R	R	R	1.56×10^3^	1.99×10^3^	<1	1.73×10^3^
FO36-5B	R	N	R	N	N	6.07×10^2^	1.04×10^3^	<1	1.21×10^3^
IT38-5RC	N	N	N	R	N	2.51×10^3^	<1	9.53×10^2^	6.93×10^2^
WY24-1RC	N	N	N	R	R	3.64×10^3^	<1	2.60×10^3^	2.77×10^3^
								Titer: I.U./ml	

“N” indicates no recombination and “R” recombination in the indicated domain.

### Analysis of Deletion Mutants of the *env* Gene

**Figure 6:**
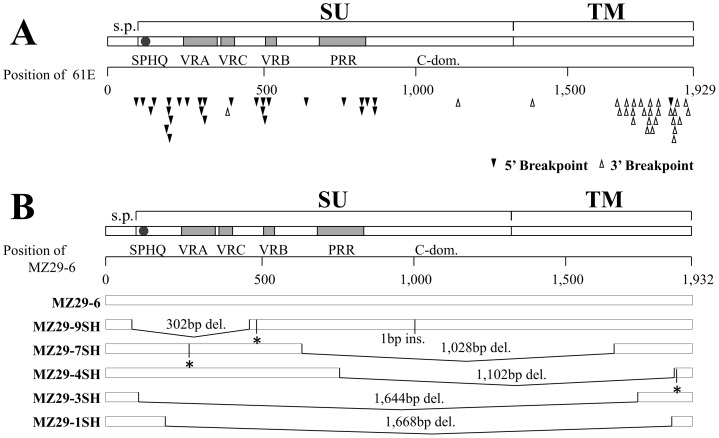
FeLV *env* gene mutants. (A) The start (5′ breakpoint) and end (3′ breakpoint) positions of segments deleted from *env* genes are indicated on the FeLV-A 61E sequence. (B) The various *env* deletion mutants isolated from sample MZ29. PCR amplifications were performed with primers Fe-8S and Fe-3R, and the product was electrophoresed and stained with ethidium bromide. Five deletion mutants (MZ29-9, MZ29-7, MZ29-4, MZ29-3, MZ29-1) and one prototype sequence (MZ29-6) are represented schematically. s.p.: signal peptide. ins.: insertion. del.: deletion. Abbreviations for specific motifs are as for Fig. 5. Asterisk indicates stop codon.

In samples from 18 cats, several smaller fragments were amplified by primer pairs Fe-8S/Fe-4S and Fe-3R, and were cloned and sequenced. Deletion regions within these *env* gene sequences were determined by comparison with full-length *env* sequences isolated from the same individual cats. The 5′ and 3′ positions of deleted regions were shown on the FeLV-A 61E *env* sequence (Fig. 6A), and several deletion patterns were evident from these plots. Multiple mutant sequences were isolated from one particular cat (MZ29), each with a different region deleted (Fig. 6B), but these sequences were all very similar to the non-mutated MZ29 *env* gene, suggesting that the various deletions could have occurred *de novo* in this particular cat. Furthermore, in the case of NR9 (see Fig. S3A), one deletion mutant of the *env* gene was isolated that had an inversion of 95-bp in addition to its deletion, and another that had a 95-bp inversion as well as an insertion of 28-bp from the cat genome sequence (see NCBI accession numbers ACBE0152516 and AANG01802278).

In addition to inversions and insertions, deletion mutants may also contain enFeLV sequences acquired by recombination, as in the case of ST17 (Fig. S3B). Overall, these results indicate that deletion events within *env* genes may often occur *de novo*, and such deletions contribute to the structural diversity of the FeLV *env* gene.

### Analysis of Structural Diversity in Small Regions of the *env* Gene

**Figure 7:**
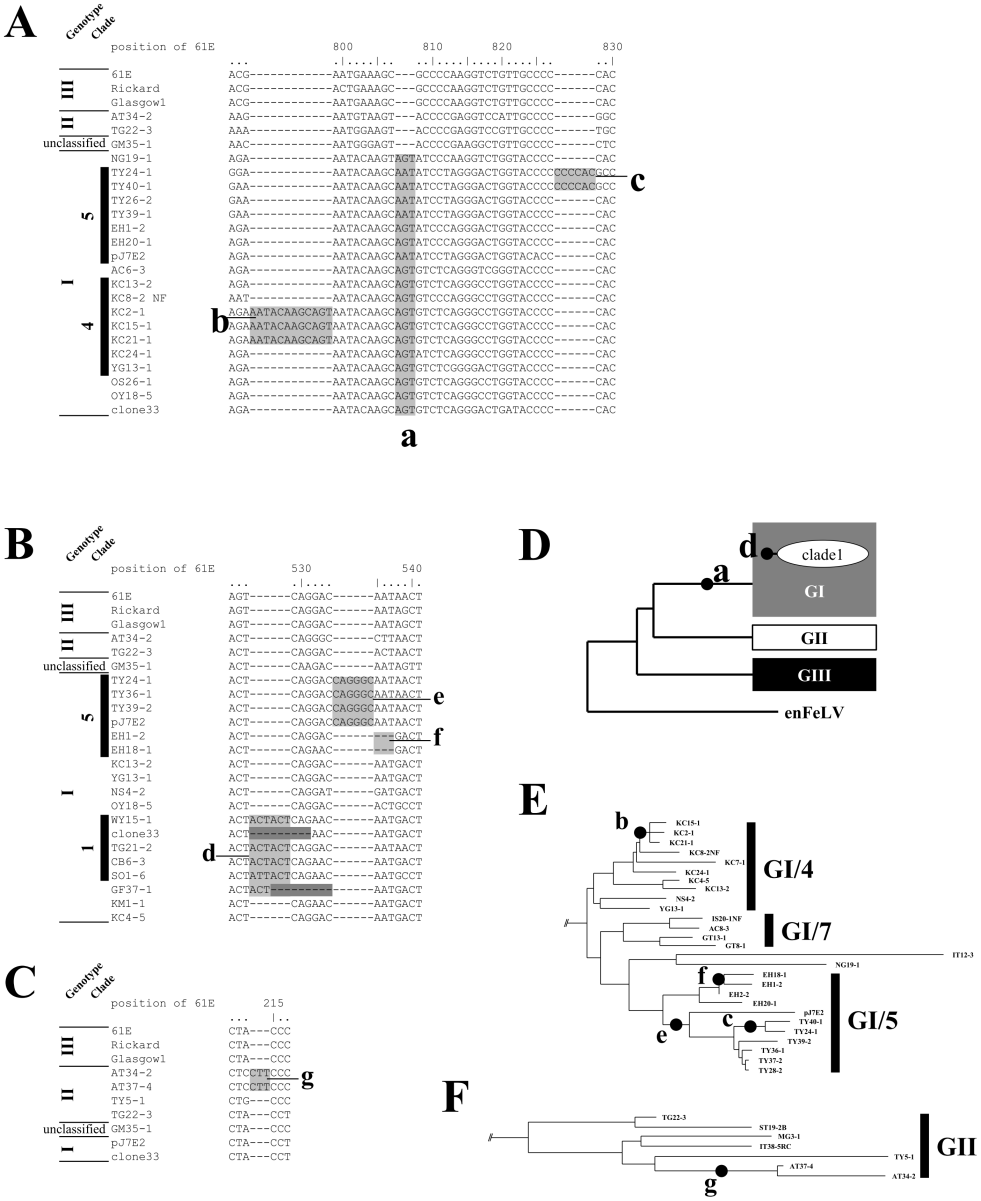
Analysis of structural diversity in small regions of the *env* genes. Characteristic indels in different versions of the *env* gene are indicated by shaded boxes and labeled with lowercase letters, shown relative to the FeLV-A 61E sequence. (A) Three insertions identified in the Genotype I group: ‘a’ – insertion of AGT or AAT; ‘b’ – insertion of AATACAAGCAGT; ‘c’ – insertion of CCCCAC. (B) Three additional indels identified in the Genotype I group: ‘d’ – insertion of ACTACT; ‘e’ – insertion of CAGGGC; ‘f’ – deletion of three nucleotides at position 535–537 of the FeLV 61E sequence. Boxes with darker shading at ‘d’ indicate atypical deletions. (C) A single insertion identified in the Genotype II group: ‘g’ – insertion of CTT. (D) – (F) show these indels (‘a’ to ‘g’) plotted on various representations of the best phylogenetic tree shown in Fig. 3.

All known full-length *env* genes observed in other countries belong to the GIII group and are 1929 bp in length, whilst FeLV *env* genes isolated from Japanese cats are variable in size. The *env* genes from GI/Clade1 are generally 1938 bp in length and *env* genes from GI/Clade2 and GI/Clade3 are typically 1932 bp in length. We searched for defining sequence characteristics for the various genotype and clade groupings, and found a 3-bp insertion (AGT/AAT) (Fig. 7A, box a) in the FeLV *env* gene of most Genotype I sequences, between positions 807 and 808 of the FeLV-A 61E sequence (Genotype III). This is absent from Genotypes II and III, and from the GM35 FeLV *env* sequence (genotype unclassified), so it seems logical to assume that the insertion must have originated in the common ancestor of Genotype I. Some clones from GI/4 have a 12-bp insertion (AATACAAGCAGT) between positions 798 and 799 of the FeLV-A 61E sequence (Fig. 7A, box b). In GI/5, a 6-bp insertion (CCCCAC) is present in isolates from TY24 and TY10, between positions 827 and 828 of the FeLV-A 61E sequence (Fig. 7A, box c), and a 6-bp insertion (ACTACT) is present in some GI/1 isolates between positions 528 and 529 of the FeLV-A 61E sequence (Fig. 7B, box d). Some GI/5 isolates include a 6-bp insertion (CAGGGC) between positions 534 and 535 of the FeLV-A 61E sequence (Fig. 7B, box e), whilst others have a 3-bp deletion (AAT) encompassing positions 535–537 of the FeLV-A 61E sequence (Fig. 7B, box f). In Genotype II, FeLVs from cats AT34 and AT37 have a 3-bp insertion (CTT) between positions 214 and 215 of the FeLV-A 61E sequence (Fig. 7C, box g). The location on the phylogenetic map (indicated as a and d in Fig. 7D, b,c,e and f in Fig. 7E, and g in Fig. 7F) of these small structural changes in the *env* gene of exogenous FeLVs indicates that these genetic traits were established in viruses and transmitted to cats via viral infection.

## Discussion and Conclusion

FeLV infection in Japan is frequently observed amongst cats that are free to venture outside. It has been shown that FeLV transmission is related to close contact between cats, such as mutual grooming or sharing food or water containers. The incidence of FeLV infection is clearly dependent in part on the region, indicating that infection may be associated with specific habits or lifestyles of either (or both) the animals or the people.

The FeLV genome was detected in the PBMC of 77.7% of the cats which tested positive for the FeLV antigen. Failure to amplify viral sequences in the remainder of infected cats indicates a certain level of inconsistency between the antigen-screening procedure and our PCR-based method. Although nested PCR amplification was carried out in these samples, we did not detect any viral genome. PBMCs infected with FeLV may not always be circulating or present in the blood, even though the cats show viremia. It may depend on the timing of collecting the blood from the cats. It has been reported that in domestic cats with FeLV-FAIDS, the virus variant FeLV/61C was mainly found in the intestines and was not present in the bone marrow [7]. Thus, different strains of the virus may target different cells or tissues, and it would be informative to determine the copy number of FeLV provirus in each tissue type in naturally infected cats. We further established a PCR method for detecting FeLV-B. This PCR method can be used as diagnostic test because the previous studies reported higher incidence of the FeLV-B variant in cats with malignant tumors or hematopoietic disorders [33], [63]. It could be used to establish the clinical stage of such diseases.

One important goal of the present study was to use sequence comparisons to investigate the structural and genetic diversity of FeLV *env* genes derived from cats that had been naturally infected with FeLV. We have previously determined the nucleotide sequences of two Japanese FeLV strains: FeLV-A clone33 isolated from cats with AML, and pJ7E2 isolated from cats with thymic lymphoma. On the basis of sequence homology, these *env* sequences were highly divergent from FeLV-A Glasgow-1 and FeLV-A 61E sequences, which prompted us to investigate the genetic diversity of the FeLV *env* gene in detail. This is the first report of a comprehensive analysis of FeLV *env* genes isolated from naturally infected cats.

The strongly supported phylogenetic structuring of the FeLV sequences analyzed in this study was unexpected. We identified three major genotypic groups, GI (containing clades 1–7), GII, and GIII (containing clades 1–2), and we found that most FeLV strains in Japan are highly associated with geographical distribution, while GI/3, GI/4 and GI5 are strongly associated. It is thus possible that these FeLV strains may have arisen by isolation in their respective areas of occurrence over a long period of time, producing a clear phylogenetic structure consistent with geographic distribution. Japanese FeLV strains are genetically distinct from variants of the virus occurring in other parts of the world, and have apparently originated and diversified within Japan. Detection of the GI/Clade 1 genotype in the geographically disjunct Kyoto, Wakayama and Oita prefectures, and detection of the GI/Clade2 genotype in the Kagoshima prefecture (geographically distant from other populations of this clade) are thought to be due to anthropogenic translocation of infected cats. With the exception of a single cat from Okinawa, FeLV sequences belonging to the GIII group were not detected in Japan, indicating that the GI and GII groupings are likely to be the dominant virus genotypes present in Japanese domestic cats. In a study of purifying selection, we did not observe significant branch-specific positive selection in Japanese FeLV strains, suggesting that adaptive evolutionary change (driven by variation in host cats, extrinsic environmental pressures, etc.) is an unlikely explanation for the observed variation in the viral *env* gene. In addition, there is no evidence that genetic diversification has led to altered receptor usage in the various strains comprising the FeLV Genotype I grouping; most epidemic FeLV strains analyzed in this study appear to belong to the FeLV-A receptor subgroup, which is thus the most prevalent subgroup among Japanese cats. In addition to genetic diversity, substantial structural diversity of the FeLV *env* gene was detected in our samples, including insertions, deletions, inversions and recombination. Similar types of structural diversity in the *env* gene (point mutations, insertions, deletions and recombination) were observed in cats that had been experimentally infected with FeLV [64]. Several FeLV *env* recombinants have also been reported in cats with naturally occurring lymphomas [33]. Retroviral recombination, such as transduction of cellular oncogenes and circulating recombinant forms (CRF), has been reported to involve ‘copy-choice’ or ‘template switching’ via formation of heteroduplexes of the viral RNA genome during reverse transcription [65], [66]. The endogenous FeLV *env* gene is expressed in normal cat lymphoid organs and FeLV-negative lymphomas [59], [67]. Therefore, exogenous FeLV infections involving these organs may expedite FeLV recombination. Analysis of recombination between exogenous and endogenous FeLVs revealed several recombination hot-spots on the *env* gene (areas in which multiple recombination breakpoints coincide); recombination tends to occur preferentially in regions of high homology between exogenous and endogenous FeLV sequences. In contrast, whilst deletion events also appear to be relatively common in FeLV strains, there may be fewer constraints on the locations of these deletions and therefore no pronounced hot-spots of deletion breakpoints on *env* gene sequences. Overall, the combined effect of recombination, insertion and deletion events is likely to result in a proliferation of structural diversity in the FeLV *env* gene.

In this study we found some unique recombinant *env* genes that contain both VRA and VRB regions derived from the endogenous FeLV *env* gene. Particular recombination patterns in *env* genes as well as point mutations affecting the SPHQ motif of the Env protein can alter viral infectivity and cell type specificity of viruses. For example, we confirmed that certain recombinant FeLV strains belong to receptor subgroups FeLV-B or FeLV-T instead of the more common FeLV-A subgroup. The SPHQ motif is highly conserved within the genus *Gammaretrovirus* and has been identified as an important determinant for mediating fusion events during virus entry into host cells [68], [69]. The FeLV-T receptor subgroup has a particular mutation (H6P) within the SPHQ motif, and this subgroup has been shown to possess unique receptor usage, involving Pit1 and FeLIX [17]. Eighteen FeLV *env* clones with mutations in the SPHQ motif were isolated from 11 samples, suggesting that the viruses belonging to the FeLV-T subgroup may be relatively common in Japan and may arise *de novo* in individual cats via mutation of FeLV-A viruses. These events are also likely to amplify viral diversity. Overall in this study, we show that the structural and genetic divergence of FeLV has probably been brought about by substitution, deletion, insertion, and recombination events within *env* genes, and these processes are thus likely to be key drivers of FeLV evolution, especially in Japan. We have identified structural and genetic diversity of FeLV *env* genes that may provide new insights into the prevention of FeLV infection, development of more effective vaccines, the geographical components of FeLV infection, and the evolutionary dynamics of the virus. Furthermore, in keeping with our division of FeLV samples into three major genotype groups and several cohesive clades within these groups, we propose that phylogenetic relationships be used as one of the methods of viral classification.

## Supporting Information

Figure S1 **The maximum-likelihood (ML) tree from phylogenetic analysis of near-full-length** ***env*** **nucleotide sequences used for dN/dS analysis.** The sequences used were listed in Supplemental Table 4. Duplicated sequences were removed.
(TIF)

Figure S2 **Branch specific selection forces for each genotype and clade.** Genotype or clade specific dN/dS ratio is shown above each root branches of the schematic phylogenetic tree. The ratio of dN/dS >1 indicates the force of significant positive selection (colored in red).
(TIF)

Figure S3 **Isolates with atypical structural mutations.** Schematic representation of the various structural mutations is shown. The motifs are abbreviated s.p. (signal peptide), SPHQ (SPHQ motif), VRA (variable region A), VRC (variable region C), VRB (variable region B), PRR (proline-rich region) and C-dom. (C-terminal domain). (A) Three clones (NR9-7, NR9-2SH and NR9-4SH) were isolated from cat NR9, and NR9-2SH and NR9-4SH had small inverted sequences (95 bp; pale gray) in addition to 1 kbp deletions. Isolate NR9-4SH also had very small inserted sequence (28 bp; black) which may be originated from a part of cat genomic sequence. (B) Two isolates from cat ST17 had FeLV-B-like recombination as well as deletion. enFeLV derived sequence are colored in black. Asterisk indicates stop codon.
(TIF)

Table S1 **Reference sequences obtained from NCBI database.**
(TIF)

Table S2 **Incidence of FeLV observed in cats in private veterinary hospitals located in each prefecture in Japan.**
(TIF)

Table S3 **Geographic distribution of each genotype of FeLV in Japan.**
(TIF)

Table S4 **The genes used in the analysis of branch-specific selection forces.**
(TIF)
