# Supplementary figures and images for: Phylogenetic and Structural Diversity in the Feline Leukemia Virus Env Gene
Source: PLoS One. 2013 Apr 11;8(4):e61009. doi: 10.1371/journal.pone.0061009 (PMC3623909; doi:10.1371/journal.pone.0061009)

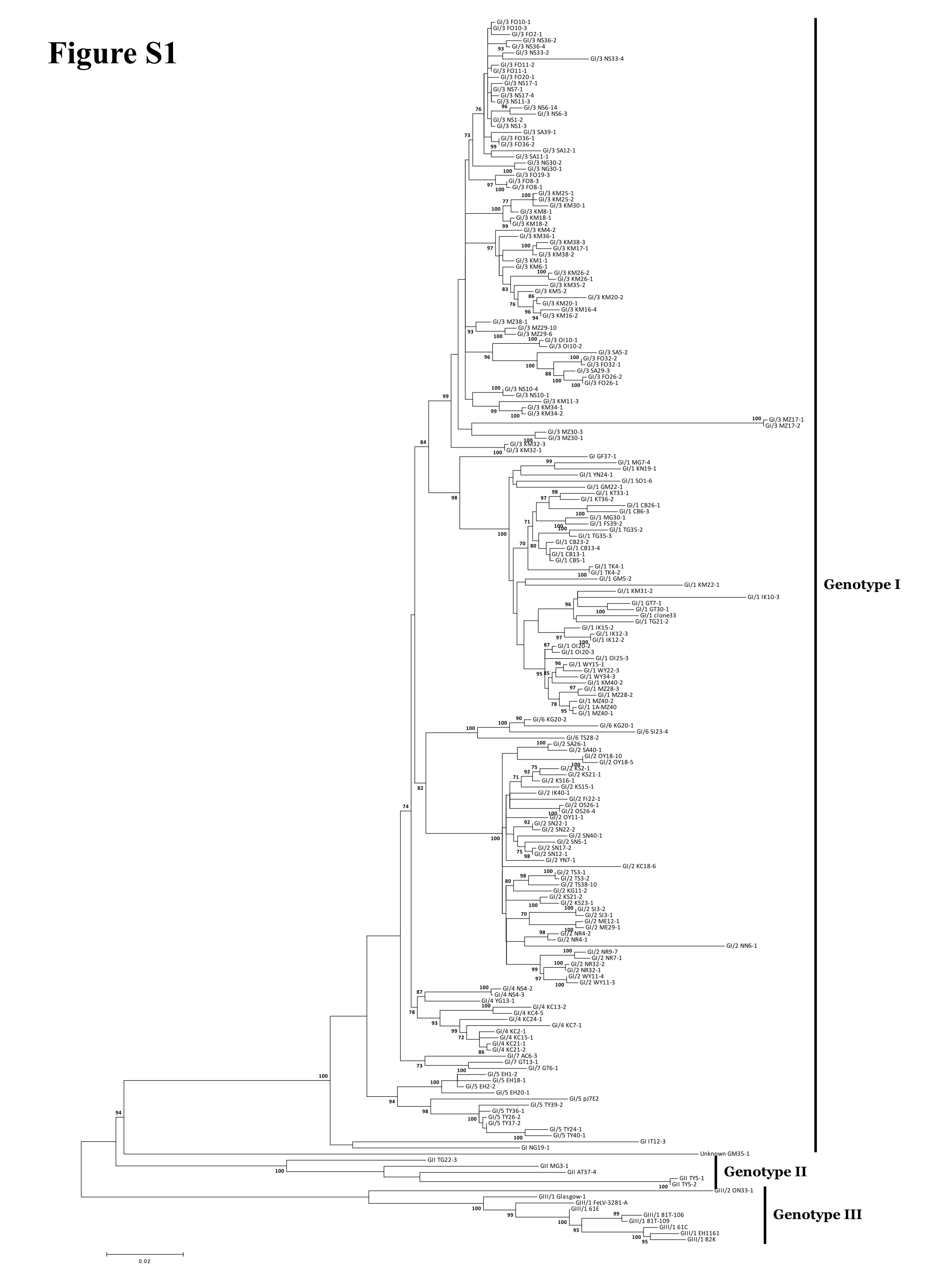

Supplement: Figure S1 — The maximum-likelihood (ML) tree from phylogenetic analysis of near-full-length env nucleotide sequences used for dN/dS analysis. The sequences used were listed in Supplemental Table 4. Duplicated sequences were removed. (TIF) [file pone.0061009.s001.tif]

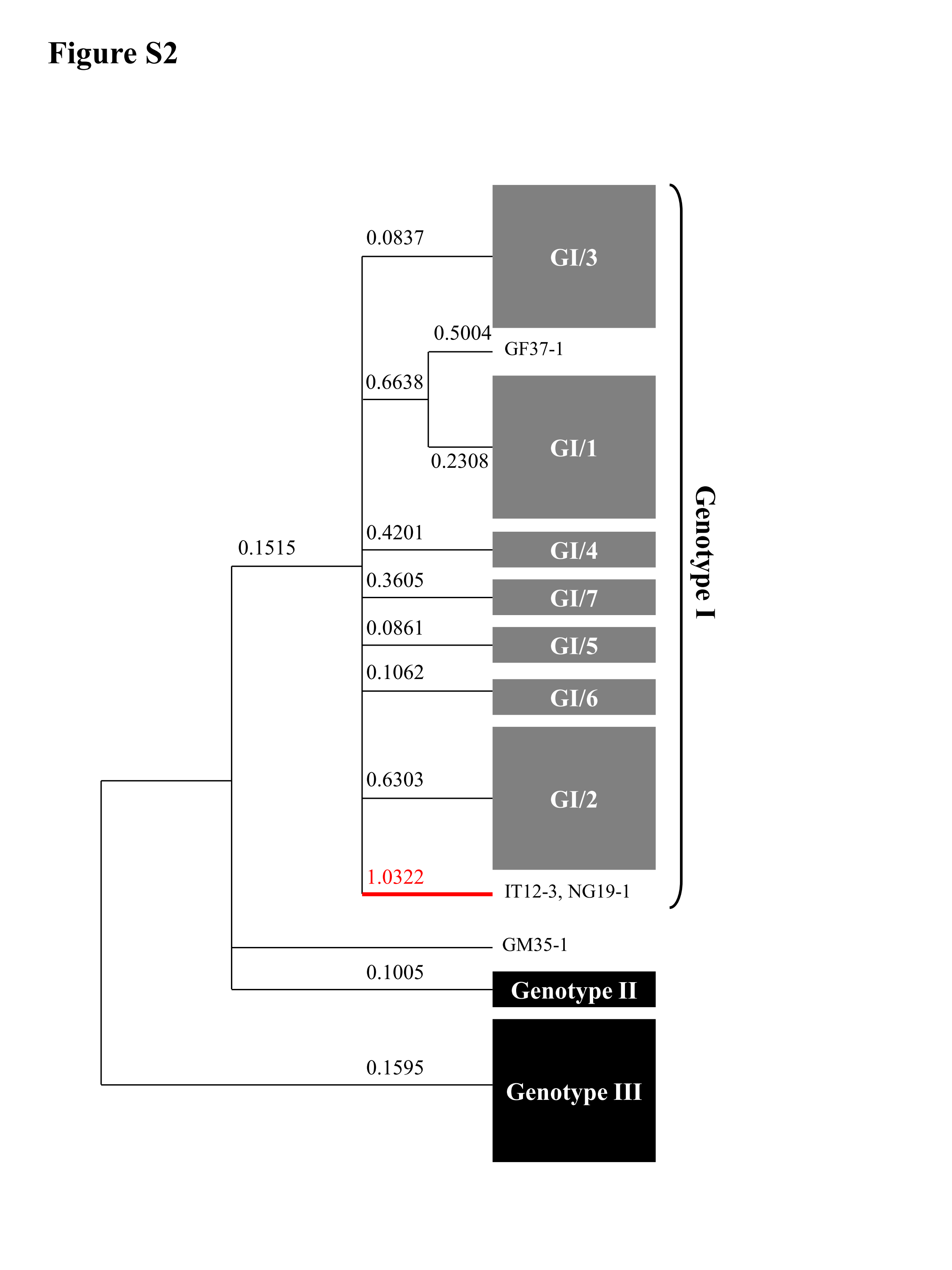

Supplement: Figure S2 — Branch specific selection forces for each genotype and clade. Genotype or clade specific dN/dS ratio is shown above each root branches of the schematic phylogenetic tree. The ratio of dN/dS >1 indicates the force of significant positive selection (colored in red). (TIF) [file pone.0061009.s002.tif]

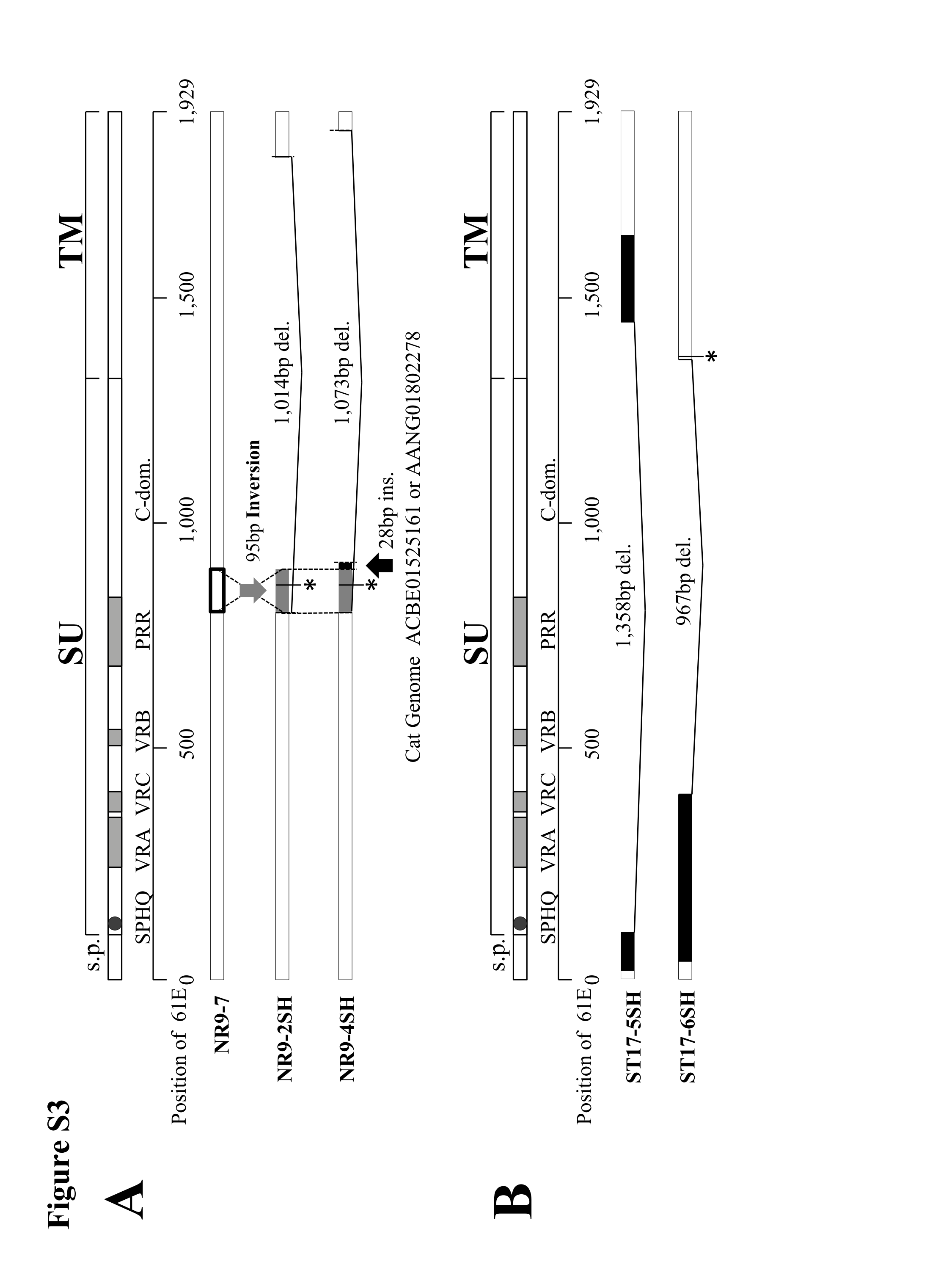

Supplement: Figure S3 — Isolates with atypical structural mutations. Schematic representation of the various structural mutations is shown. The motifs are abbreviated s.p. (signal peptide), SPHQ (SPHQ motif), VRA (variable region A), VRC (variable region C), VRB (variable region B), PRR (proline-rich region) and C-dom. (C-terminal domain). (A) Three clones (NR9-7, NR9-2SH and NR9-4SH) were isolated from cat NR9, and NR9-2SH and NR9-4SH had small inverted sequences (95 bp; pale gray) in addition to 1 kbp deletions. Isolate NR9-4SH also had very small inserted sequence (28 bp; black) which may be originated from a part of cat genomic sequence. (B) Two isolates from cat ST17 had FeLV-B-like recombination as well as deletion. enFeLV derived sequence are colored in black. Asterisk indicates stop codon. (TIF) [file pone.0061009.s003.tif]

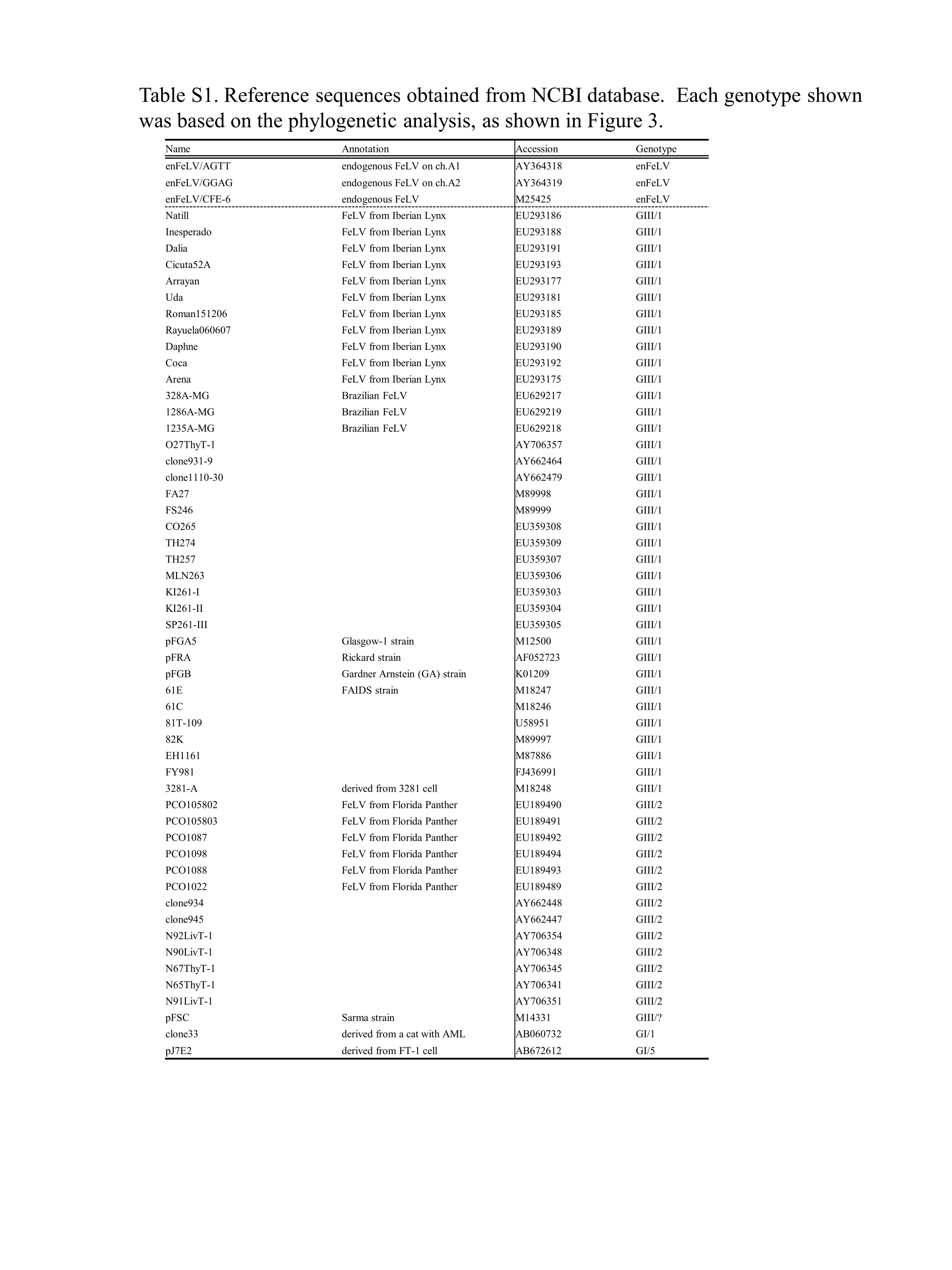

Supplement: Table S1 — Reference sequences obtained from NCBI database. (TIF) [file pone.0061009.s004.tif]

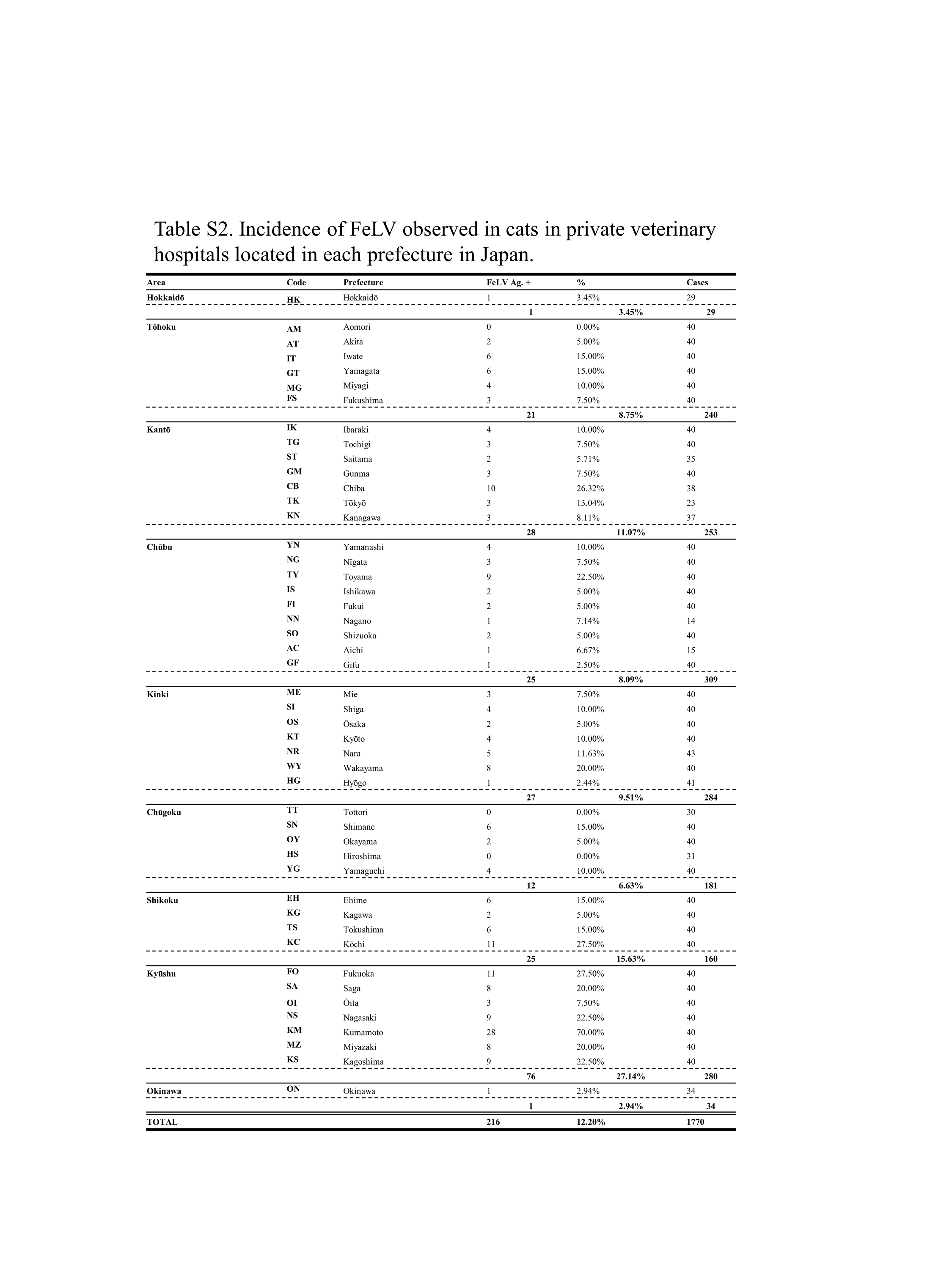

Supplement: Table S2 — Incidence of FeLV observed in cats in private veterinary hospitals located in each prefecture in Japan. (TIF) [file pone.0061009.s005.tif]

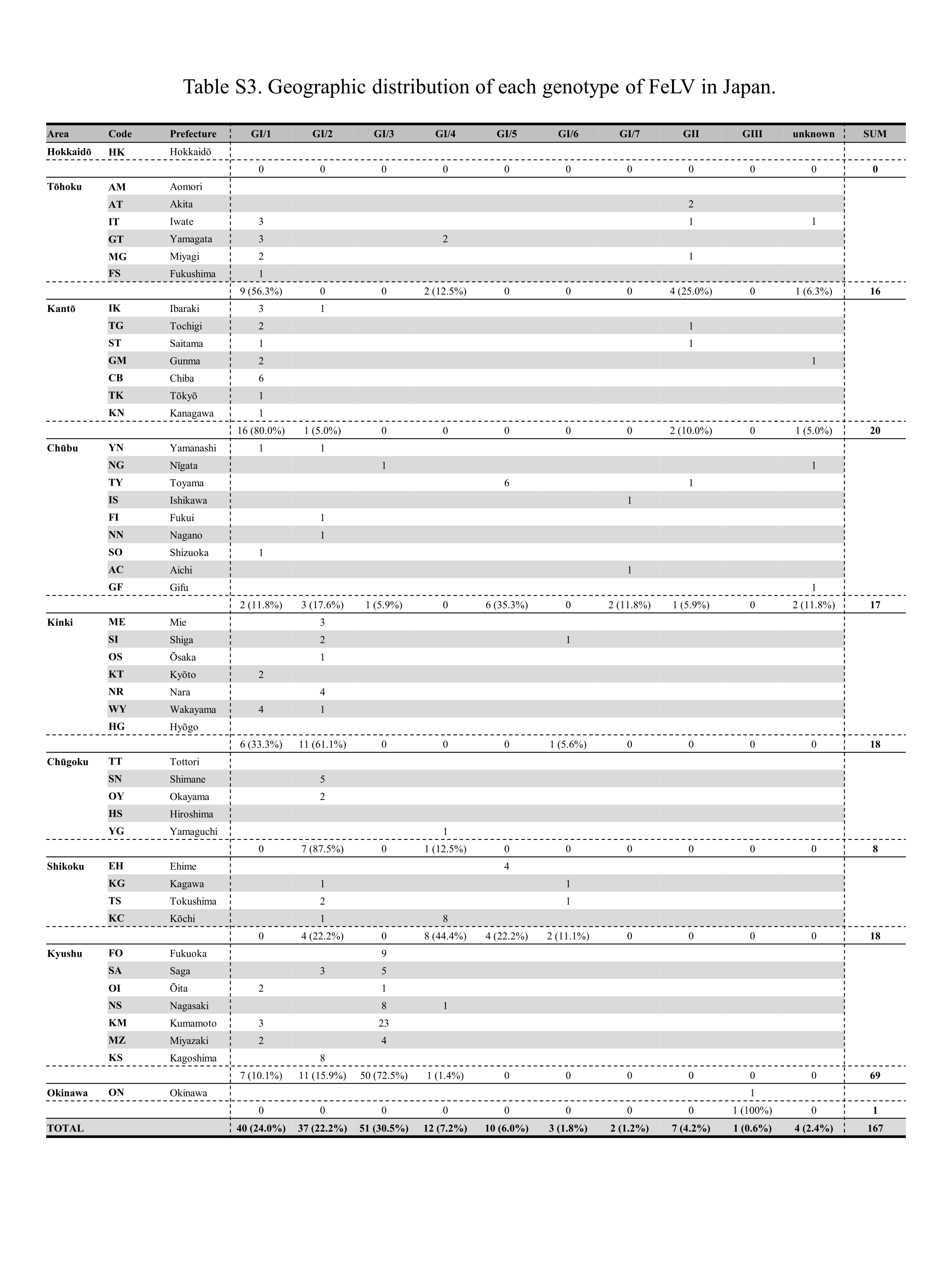

Supplement: Table S3 — Geographic distribution of each genotype of FeLV in Japan. (TIF) [file pone.0061009.s006.tif]

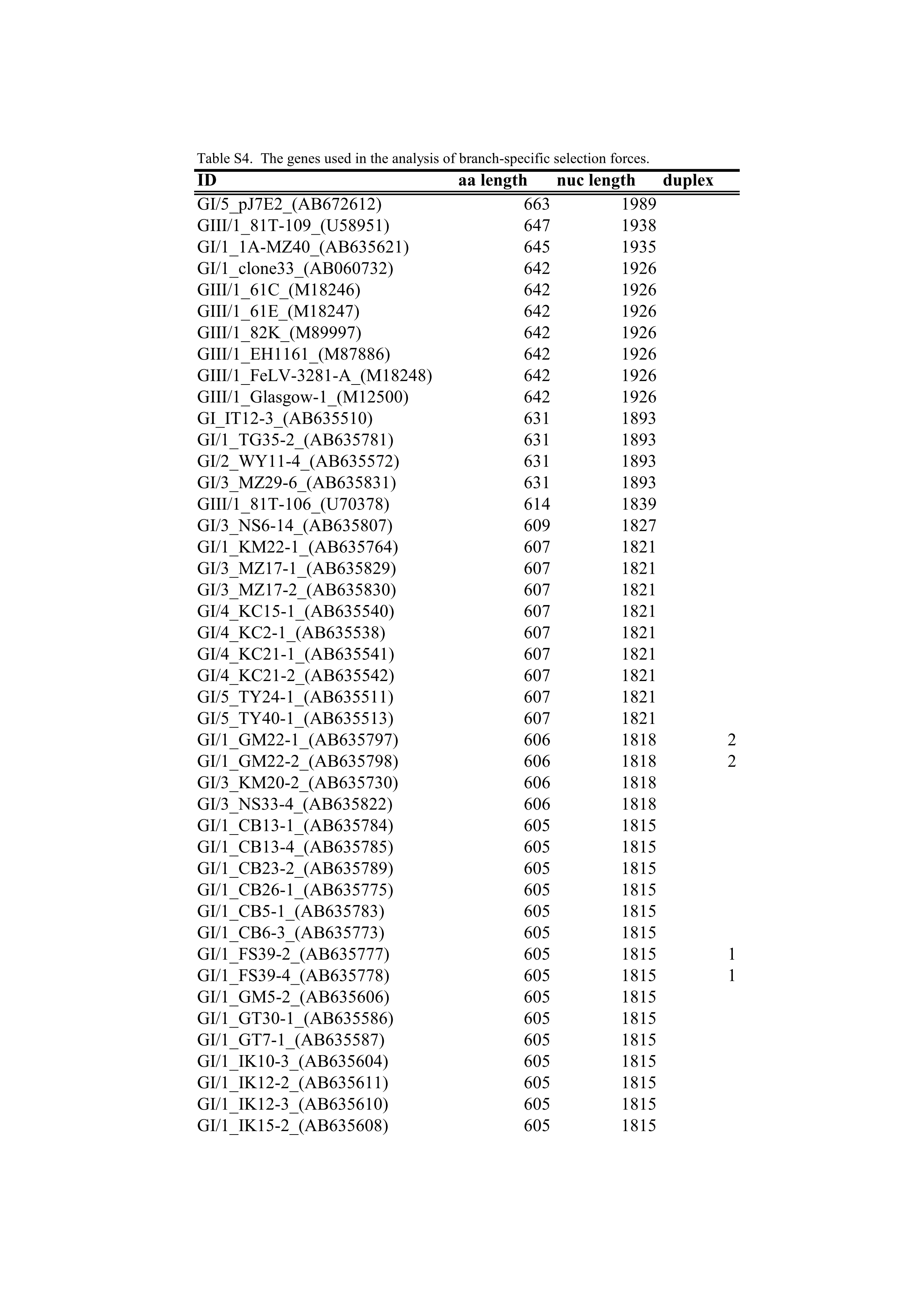

Supplement: Table S4 — The genes used in the analysis of branch-specific selection forces. (TIF) [file pone.0061009.s007.tif]
